# The effect of natural fillers on the marine biodegradation behaviour of poly(3-hydroxybutyrate-co-3-hydroxyvalerate) (PHBV)

**DOI:** 10.1038/s41598-020-78122-7

**Published:** 2021-01-13

**Authors:** Kjeld W. Meereboer, Akhilesh K. Pal, Erick O. Cisneros-López, Manjusri Misra, Amar K. Mohanty

**Affiliations:** 1 School of Engineering, Thornbrough Building, University of Guelph, 50 Stone Road East, Guelph, ON Canada; 2 Bioproducts Discovery and Development Centre, Department of Plant Agriculture, Crop Science Building, University of Guelph, 50 Stone Road East, Guelph, ON Canada

**Keywords:** Environmental impact, Engineering, Materials science

## Abstract

Worldwide, improper disposal of plastics is instigating environmental initiatives to combat plastics accumulation of in the environment and the world’s oceans. Poly(3-hydroxybutyrate-co-3-hydroxyvalerate) (PHBV) biocomposites with *Miscanthus* (Misc) fibres and distillers’ dried grains with solubles (DDGS) were studied to ascertain if natural fibres and proteinaceous fillers can improve polyhydroxyalkanoate marine biodegradability. Using ASTM standard D7991-15, the biodegradation of PHBV, PHBV with Misc (15 and 25 wt%) and PHBV with DDGS (15 and 25 wt%) was performed in a simulated marine environment for the first time, as indicated by a literature survey. PHBV/Misc (85/15) and (75/25) biocomposites showed 15 and 25% more biodegradation compared to PHBV, respectively. Proteinaceous PHBV/DDGS (85/15) and (75/25) biocomposites showed 17 and 40% more biodegradation compared to PHBV, respectively. Furthermore, PHBV/Misc (75/25) and PHBV/DDGS (75/25) biocomposites were marine biodegraded in 412 and 295 days, respectively. In conclusion, proteinaceous fillers (DDGS) biocomposites have better marine biodegradability than *miscanthus*.

## Introduction

In the evolving economy, bio-based plastics are making their mark and considered part of the new economy encompassing recycled commodity plastics^1^. The shifting in economic objectives is driven by the expected cumulative growth of main plastic waste which will surpass 25 billion tonnes by 2050^2^. Currently, 7.7 million tonnes of plastic waste leak into marine environments from land every year, which either become inaccessible if sunk or travel to other shores^3^. The impact of ocean plastic waste is more concerning for the ecosystem, as various species have been found to have consume it. It’s predicted 99% of all seabirds will have ingested plastic waste by 2050, which can impact gut performance and fledgling survival rates^4^. Therefore, there is a need for biodegradability of plastics in marine environments, which may be possible when biodegradable plastics can replace current plastic articles and reduce the impact on the future ecosystems.

Biodegradation of a polymer can be defined as a bio-fragmentation process (including changes in its chemical structure and properties) followed by assimilation by microorganisms (fungi, algae or bacteria), through their metabolic activity under specific conditions^5^. During biodegradation, biopolymers are disintegrated and converted into carbon dioxide or methane, water and biomass in compost, landfills, soil, marine or other environments. For example, a major mode of degradation for polyhydroxyalkanoates (PHAs) is through enzymatic hydrolysis^6^. Such biodegradable polymers hold a significant advantage in reducing the future potential plastic waste leakage and retention in the environment, with the intention of letting the plastics degrade rapidly. However, it should be coupled with a bio-based method of production to reduce greenhouse gas emissions, for which it is important to integrate the precepts of the circular bioeconomy and apply biodegradability assessments and life cycle analysis.

It has been found that renewable plastics have a significantly lower energy requirement during production and a significantly lower carbon emission at end of life^7^. Therefore, bio-based biodegradable polymers have the potential to combat climate change with sustainable production methods and reduced impact after disposal. The most common bio-based biodegradable polymers utilized in industry are cellulose acetate, poly(lactic acid) (PLA), thermoplastic starch based blends and PHAs^8^. However, it is well accepted that biodegradation is selective, depending on several factors such as the physical and chemical properties of biopolymers, as well as on the conditions of the biodegradation media and, consequently, on the presence and availability of microorganisms capable of carrying out the process. For example, a biopolymer that shows biodegradability under aerobic conditions in liquid media will not necessarily be biodegradable under other conditions, due to the fact that a bacterial community will not be able to widely biodegrade all kinds of biopolymers, leading to a possible accumulation in the environment when these materials are used extensively^5^.

For this reason, it is very important to carry out studies to identify the appropriate biodegradation medium for each biopolymer. Industrial composting (along with landfills and anaerobic digesters) is one of the main large scale methods of biodegradation. It is a well-controlled process where biopolymers such as cellulose acetate^9^ and PLA degrade^10^, although outside of the industrial composting conditions they show little if any degradative behaviour. Composting takes place at 55–60 °C, compared to the natural environment which may occasionally exceed 30 °C. Therefore, if these polymers reach the environment, e.g. the ocean, their fast biodegradation is not warranted. In order to truly minimize the plastic waste environmental impact, PHAs and thermoplastic starches are an ideal solution in this case.

PHAs are the most well-known marine biodegradable biopolymers, which are suited to reducing the long-term impact of ocean plastic waste. PHAs are degraded by a large number of bacteria and fungi^11^. Poly(3-hydroxybutyrate-co-3-hydroxyvalerate) (PHBV) is a copolymer based on homopolymer poly(3-hydroxybutyrate) (PHB), with hydroxyvalerate units that increase the amorphous fraction, improving the mechanical performance and biodegradability compared to PHB^6^. Compared to other biodegradable polymers (i.e. poly(ε-caprolactone) (PCL), poly(ethylene adipate) (PEA), poly(ethylene succinate) (PES) etc.), PHBV can be degraded in both fresh and saltwater within 28 days provided the microbial activity and environmental conditions are suitable. The complete biodegradation of other PHAs such as PHB and poly(3-hydrobutyrate-co-4-hydroxybutyrate) (PH4B) take slightly longer^12^. According to some experiments available in the literature, PHBV also shows biodegradable behaviour in soil, water, or sand, or a combination of these, which suggests a wide distribution in the environment of microbes capable of degrading PHA^13^. Thus, it is well established that PHAs and, by virtue of being a subset of PHAs, PHBV are biodegradable in most natural environments.

A study of the lifetime of PHA products in the natural marine environment was conducted from the available literature, and it was concluded that a PHA water bottle may take 1.5–3.5 years to degrade completely in the marine environment^14^.

Typically, PHBV is expensive to purchase, due to its production costs and poor availability in industry^8^. Therefore, price reduction is significantly important for mass production and adoption in commodity applications. The blending of PHAs with biodegradable polymers (i.e. PLA, poly(butylene adipate terephthalate) and poly(butylene succinate) can slightly reduce costs, but is usually undesirable as it may impact the relative biodegradability or the environmental sustainability due to petroleum resource requirements. Biomass fillers from the agroindustry, such as natural fibres, have been incorporated into polymer matrices to reduce costs and increase performance in automotive applications. Therefore, PHA biocomposite adoption can further benefit from the research on improvement of the biodegradability and sustainability of PHAs. Natural fibres and fillers are mainly composed of cellulose, hemicellulose and lignin, which have been reported to improve the biodegradation of PHAs in aerobic environments^15–17^. However, major studies are based on soil and compost environments. Using ASTM D6691-17, PHBV/CaCO_3_ and 20% seagrass fibre composites have shown improved biodegradation rates due to poor matrix-fibre interfacial adhesion which improved physical degradation and enhanced biological attack^18^. Similarly, incorporation of starch into PHBV has shown biodegradation rate improvements in natural marine water over PHBV alone^19^. Marine biodegradation of PHA-based biocomposites remains relatively unexplored.

Furthermore, protein containing natural fibres and fillers provide an additional attribute that increases the complexity of biodegradation. Distillers’ dried grains with solubles (DDGS), a waste from corn bioethanol production, contains 30% protein before any treatment^20^, which includes beneficial forms of nitrogen that can be used by microorganisms. During thermal processing, protein denaturation and degradation occur, releasing several compounds trapped in the polymer matrix and fibres, mainly consisting of CO_2_, NH_3_, H_2_O and other sulphides^21^, which are exposed to the environment during biodegradation. NH_3_ naturally forms a chemical equilibrium with NH_4_^+^ in the presence of water, which can be used as a nitrogen source. Furthermore, ammonia can also be absorbed and utilized by microorganisms in the nitrogen cycle as nitrates after ammonia oxidation and nitrite oxidation^22^. Other forms of thermal degradation products and any residual protein are depolymerized by microorganisms, passed through the cellular membrane and used to form amino acids. The potential of PHBV/DDGS based biocomposites has not been explored in marine biodegradation, however, PHA/soy protein isolate and PHA/DDGS biocomposites have been found to have improved or comparable soil biodegradation properties to those of PHA/starch biocomposites at similar filler ratios^17^. PHA/DDGS and PHA/soy protein isolate have also proven successful in composting studies, showing complete degradation in 84 days^16^. Therefore, it is important to evaluate the potential effects of protein-based fibres/fillers compared to traditional fibres/fillers in marine biodegradation to combat plastic waste pollution in the world’s oceans.

The major limitation of these marine degradation studies is either the study ending before biodegradation is complete, modifications to the standard method used, or not applying a standard in any form. The marine biodegradation standard, ASTM D7991-15, involves testing polymer samples in a laboratory using natural marine water sourced from the environment (unlike ASTM D6691-17). This method allows for testing samples under conditions reflecting either floating plastics or plastics submersed in sediment in coastal waters. Additional control parameters are light exposure and sample form (ground or solid).

In this work, the main objective was to analyse the effect of two different fillers on the marine biodegradability of PHBV-based biocomposites following ASTM D7991-15 standard. The sustainable biocomposites were prepared using 15 and 25% wt. of lignocellulosic (*Miscanthus*) or proteinaceous (DDGS) fillers from the agroindustry using PHBV as a matrix. In addition to providing an alternative for reuse and valorization of agro-industrial waste, the formulation of biocomposites allows the production of greener and lighter materials, suitable for commodity applications at a lower cost with enhanced marine biodegradability and mechanical performance, which can further accelerate the carbon–neutral cycle.

## Results and discussion

### Mechanical properties

**Figure 1:**
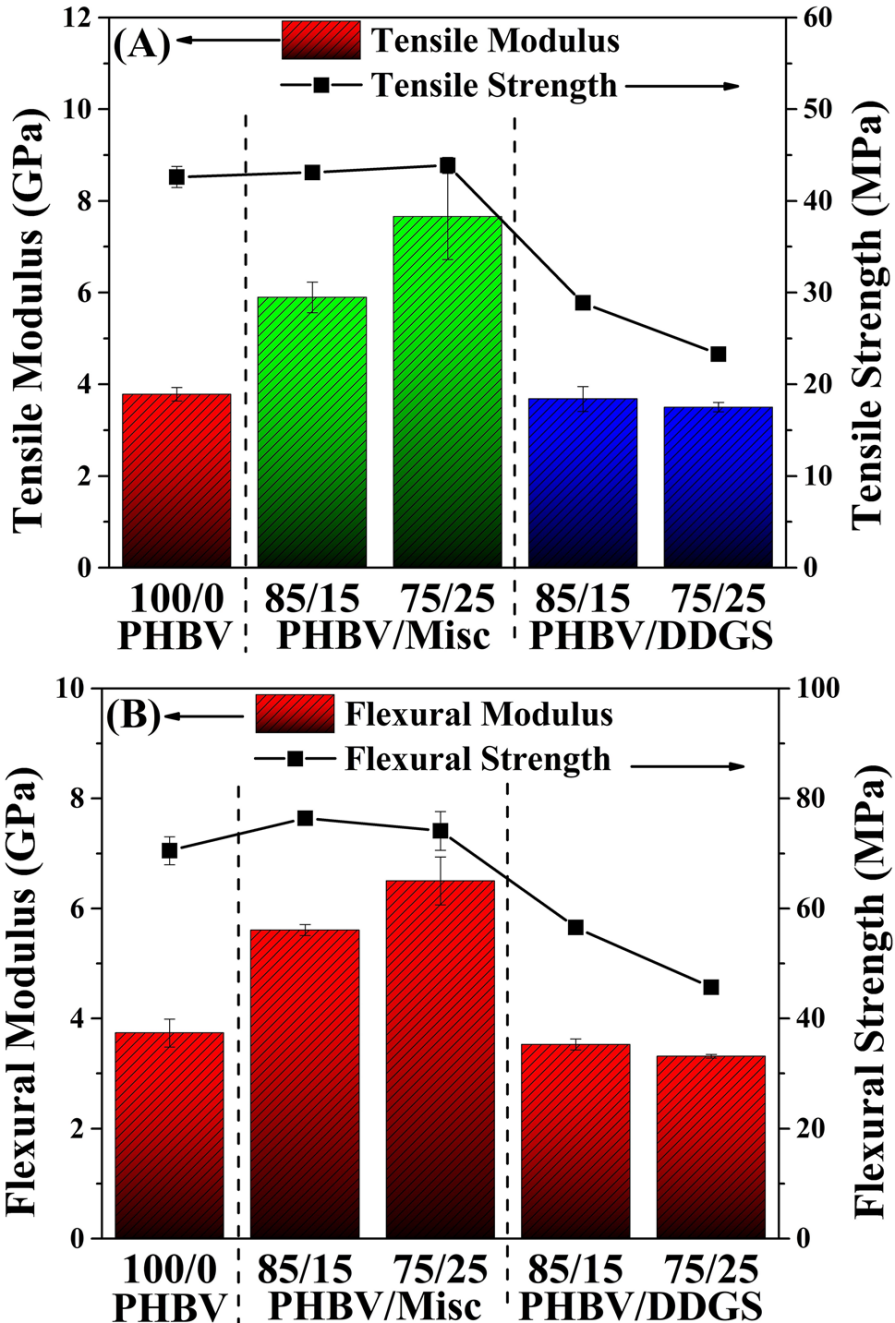
PHBV, PHBV/Misc and PHBV/DDGS. (**A**) Tensile modulus and strength, (**B**) flexural modulus and strength.

PHBV/Misc composites had increased tensile and flexural moduli by 55 and 100% relative to virgin PHBV (Fig. 1A,B) which can be attributed to the high modulus of a single internode of Misc grass (12.1 GPa)^23^. Furthermore, it was found that 15% and 25% Misc fibre increased the impact strength of PHBV (~ 13 J/m) to ~ 27 and ~ 29 J/m, respectively. However, these attributes are the result of PHBV requiring toss correction due to the impact strength being below 27 J/m. The value of PHBV samples without toss correction was ~ 21 J/m, still below that of PHBV/Misc biocomposites. Natural fibres reinforcement has been found to increase the impact strength of PHBV and some other polymers, however, it is dependent on the fibre orientation, composition, type and interfacial adhesion between the matrix and fibres^24^. The Misc fibre impedes crack propagation, which resulted in the improved impact strength. The long fibres are stronger than PHBV, requiring energy to be absorbed as they are pulled out of the PHBV matrix.

Tensile and flexural strength of PHBV/DDGS (85/15) and (75/25) biocomposites were reduced by 30 and 40%, respectively, while the tensile and flexural moduli remained unchanged (Fig. 1A,B, and Supplementary Table S1). The reinforcement effect of DDGS in PHBV/PBS blends was reported to have similar effects^25^. There was no change in PHBV/DDGS biocomposite impact strength compared to virgin PHBV. DDGS is a multi-layered grain-based filler with a low aspect ratio compared to long Misc fibres. Furthermore, DDGS has incredibly poor mechanical properties with a strength and modulus of 0.3–0.5 MPa and 2.41–5.24 MPa, respectively^26^, thus it is a poor reinforcing agent relative to Misc grass natural fibres.

### DSC

**Figure 2:**
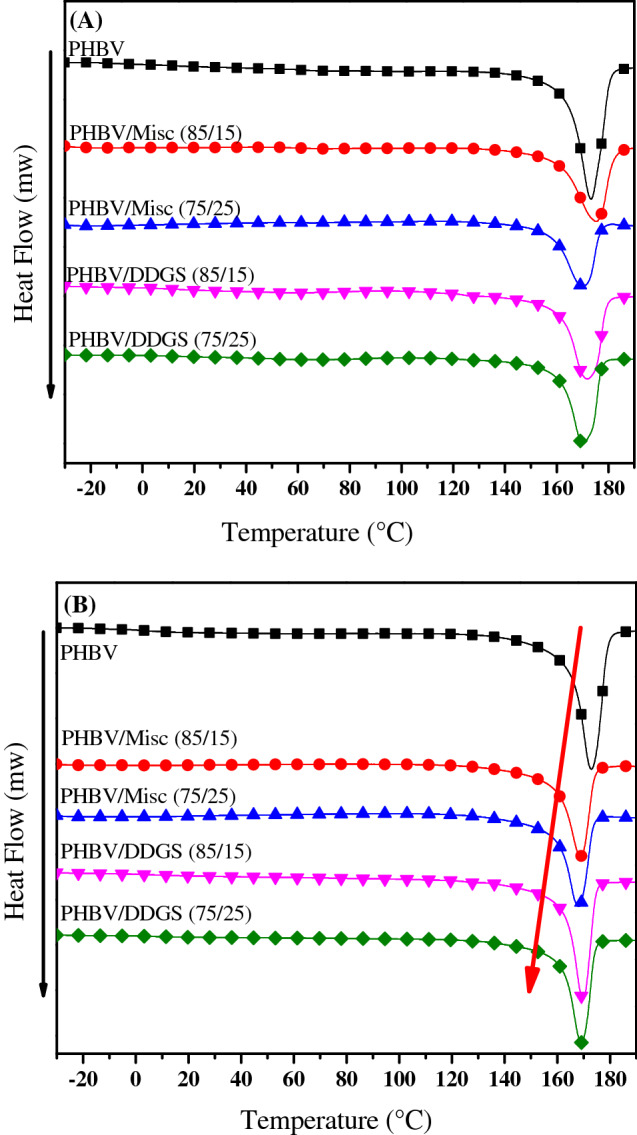
DSC (**A**) first heating cycle, and (**B**) second heating cycle of PHBV, PHBV/Misc and PHBV/DDGS biocomposites.

From the DSC curves in the first heating cycle, it was possible to identify the amorphous fraction of PHBV in the biocomposite, which is more susceptible to enzymatic and moisture penetration during biodegradation (Fig. 2A). The second heating cycle indicates a definitive melt temperature reduction with increased fibre loading, however, no change is seen between 15 and 25% loading with Misc or DDGS (Fig. 2B).

The melt enthalpy and crystallinity in the first and second heating cycles of PHBV samples (see Supplementary Table S2) agree with literature (68 and 72% respectively). Deroiné et al. reported that crystallinity of PHBV from the first heating cycle is slightly lower than from the second, correlating with the thermal properties of PHBV found here^27^. Crystallinity of Misc composites remains unchanged or slightly increased but no significant nucleating effect was found, reflecting similar results in literature^28^. It was predicted that, due to the smaller particle size of DDGS compared to Misc grass fibres, it would reduce the amorphous fraction of PHBV in the biocomposites by acting as a nucleating agent^29^. However, PHBV/Misc composites had slightly higher crystallinity, although overall the increase is insignificant.

The crystallinity in the first heating cycle is a product of the processing conditions and indicates the amorphous and crystalline PHBV fraction that microorganisms and their enzymes will interact with. The crystalline fraction provides a suitable enzyme binding site for PHA degradation^30^. However, reduced crystallinity of PHAs through processing techniques have been reported to improve enzymatic degradation kinetics, but the rate of biodegradation is reported unchanged^31^. He et al.^32^ found amorphous polymer inclusion into PHB, improved the enzymatic degradation rate of the PHB. Lignin, and starch are both examples of amorphous or partially amorphous polymers. Thus, the improved biodegradation of the biocomposites can be attributed to the presence of the natural fibres and filler composition, morphology, interfacial adhesion between the matrix and filler, and the matrix morphology.

### TGA

The onset of degradation at 10% and the peak degradation temperature of PHBV (286 °C and 302 °C) in Supplementary Table S3, are within literature values^20^. Misc fibre has a broad degradation peak (200–500 °C) associated with its cellulose and hemicellulose content but individual components could not be identified. Thus, the thermal stability of PHBV/Misc composites is reduced to 285–292 °C due to hemicellulose sensitivity to heat treatment^21^, which initiates before PHBV degradation.

DDGS has two peaks associated with the hemicellulose and cellulose, constituting approximately 20 and 29% of the DDGS, similar to those reported in literature^33^. The thermal stability of DDGS biocomposites was reduced to 287–297 °C, however, this is due to the protein sensitivity to heat in addition to the hemicellulose^21^ that initiates before PHBV degradation.

The ash content of all biodegradation samples is within 1–2%, except for cellulose which is of entirely organic and has no ash (see Supplementary Table S3). The ash content of DDGS is similar to reports in literature^33^ and, by incorporating it into PHBV, the inorganic content increased by < 1%.

The sediment contains 59% inorganic content, indicating non-carbonaceous minerals, while the organic content is composed of the carbon, hydrogen, nitrogen and oxygen elements. From the ash content, it is hypothesized that the reference sample, PHBV, PHBV/Misc and PHBV/DDGS biocomposites will degrade by approximately 98–100%.

### Elemental analysis

The cellulose elemental constituents (see Supplementary Table S4), carbon (44%) and hydrogen (6.4%), are in agreement with the theoretical cellulose composition. The residual oxygen is approximated as 49%, assuming the cellulose is entirely pure. Based on PHBV having approximately 2% hydroxyvalerate, the theoretical carbon, hydrogen and oxygen masses are 55.9, 7.0 and 37.1% respectively, in agreement with the elemental analysis results (see Supplementary Table S4).

The carbon mass (%) of Misc fibres is approximately 47–49%^34^, and the difference in hydrogen content (6%) of cellulose, hemicellulose and lignin is negligible^35^. Hence, PHBV/Misc composite elemental analysis does not differ significantly (± 1%) from PHBV. Nitrogen content is < 1%, indicating a negligible nitrogen source.

The carbon, hydrogen, nitrogen, sulphur and oxygen of DDGS are reported as 43.8, 6.8, 5.4, 1.0 and 34.2%, respectively^36^, which is expected to result in 54.5% carbon and 5.5% hydrogen in DDGS composites. PHBV/DDGS (85/15) and (75/25) had 6.7 and 7.6% nitrogen, respectively. The sediment contains approximately 11% carbon, while the residual compounds are made up of hydrogen, oxygen and any inorganic material. From the TGA and elemental analysis, the oxygen content in sediment is approximately 40%.

### SEM morphology

**Figure 3:**
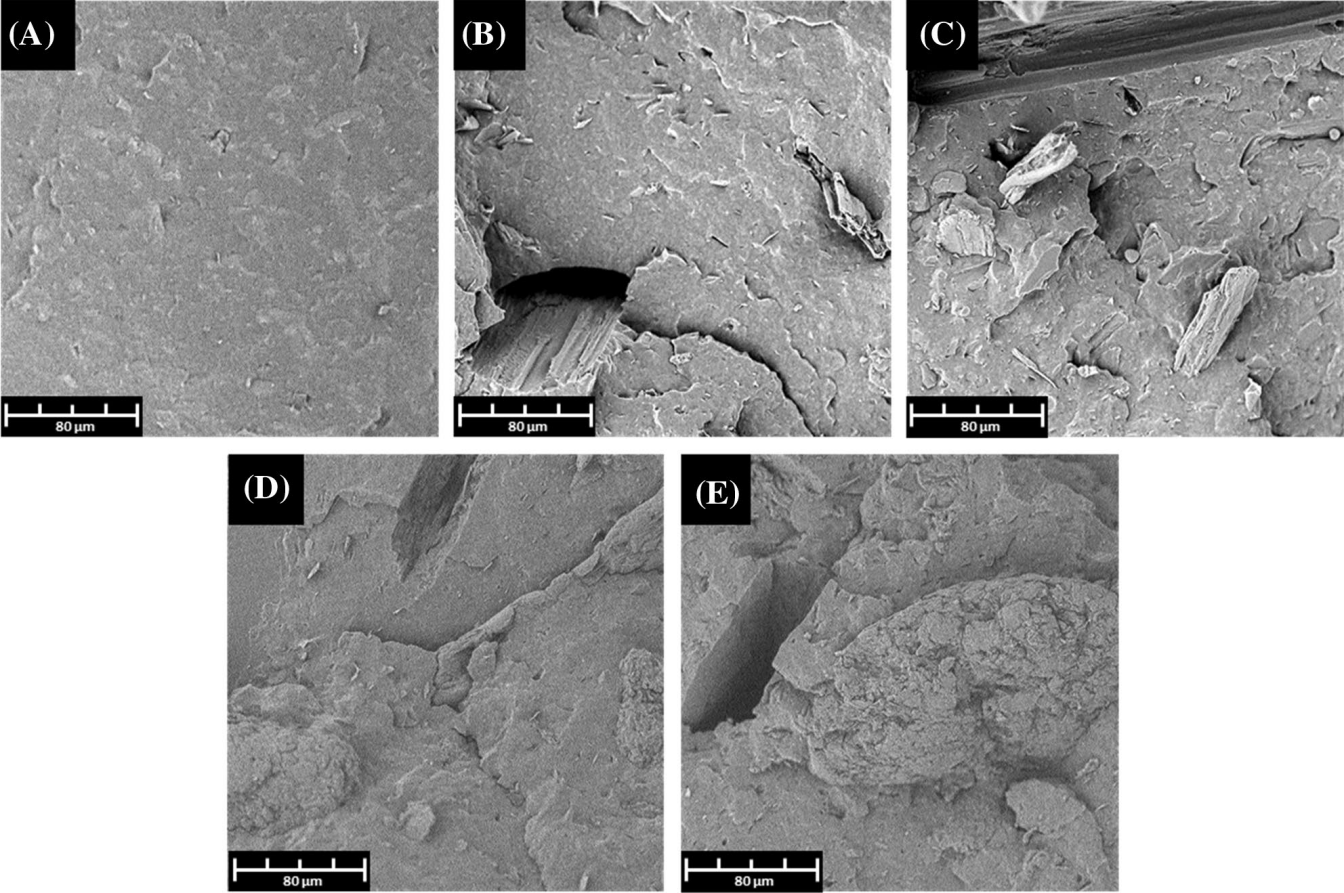
SEM morphology of (**A**) PHBV, (**B**) PHBV/Misc (85/15), (**C**) PHBV/Misc (75/25), (**D**) PHBV/DDGS (85/15) and (**E**) PHBV/DDGS (75/25) notched IZOD impact samples after breaking.

SEM morphology of impact fractured PHBV sample (see Fig. 3A) shows a smooth, undamaged, homogeneous surface. Misc fibres in the PHBV/Misc biocomposites are distributed as layered fibres throughout the matrix (Fig. 3B,C). There is some evidence of Misc fibre pull-out, which may have resulted in the increase in impact properties. The long fibres have sustained their structural integrity and, with pull-out, channels can be made throughout the polymer matrix, exposing more enzyme activity sites on the PHBV surface. Furthermore, when fibres break, they provide exfoliated carbon sources, which enhance the fibre degradation processes. DDGS inclusion in PHBV results in multiple small particles distributed throughout the structure (Fig. 3D,E). Minor evidence of DDGS pull-out is observed, however, the particles are small, and thus not expected to improve the impact properties. Furthermore, the DDGS particles have a rough surface and there is evidence of particle breakage occurring, indicating the poor mechanical strength of DDGS. Guttierez-Wing et al.^37^ reported improved PHB surface area results in increased degradation kinetics, and a direct correlation is also observed in PBS^38^. Therefore, the morphology of PHBV biocomposites after breakage show increase the surface area which can improve the degradation of samples disposed of in the marine environment.

### Contact angle

The contact angle of cellulose using both solutions was zero due to the high porosity, because the original form is filter paper as required by ASTM D7991-15 standard (see Supplementary Table S5). The contact angles of PHBV using deionized water (66.7°) and diiodomethane (39.1°) are in agreement with literature^39^. With the introduction of Misc fibre, it appears the hydrophilicity (~ 43°) and hydrophobicity (~ 73°) increased, however, the surface roughness increased, impacting the sessile drop measures of the diiodomethane due to its low surface tension. Therefore, the contact angle of PHBV/Misc composites did not provide clear results. Wu et al. has documented PHA biocomposites and, in all cases, the water absorption rate increased with increasing load of natural fibres^15^. However, surface roughness does increase surface area, which may improve the biodegradation results and indicates there are some Misc fibres on the surface of the polymer.

With the introduction of DDGS, there appears to be a slight increase in surface hydrophobicity (37.3°), however, this characteristic is only present at 25% DDGS loading. This can be attributed to the high protein component which may be hydrophobic and may reduce the water permeation and biodegradation rate. However, literature reports DDGS to significantly improve the water absorption of PHBV^20^. DDGS is also known to have 10% fat, which can migrate to the polymer matrix during processing and result in higher surface hydrophobicity^20^. Therefore, water absorption and penetration in PHBV/DDGS biocomposites is not only based on the surface hydrophilicity but on other characteristics such as the DDGS porosity and its composition.

### Marine biodegradation

Following ASTM D7991, the sample was tested in powder form, which limits the evaluation of marine biodegradation to only one characterization. If a film sample was used, the biodegradation, characterizations such as SEM and mass loss could supplement the marine biodegradation determination. However, additional steps may also interfere with the marine biodegradation as outlined by the standard and would also require more concurrent samples to be completed.

The total organic carbon (TOC) of the sediment (0.09%) was found to remain approximately the same before and after biodegradation. The marine water TOC was 1.8 mg/L, and considered to be negligible.

**Figure 4:**
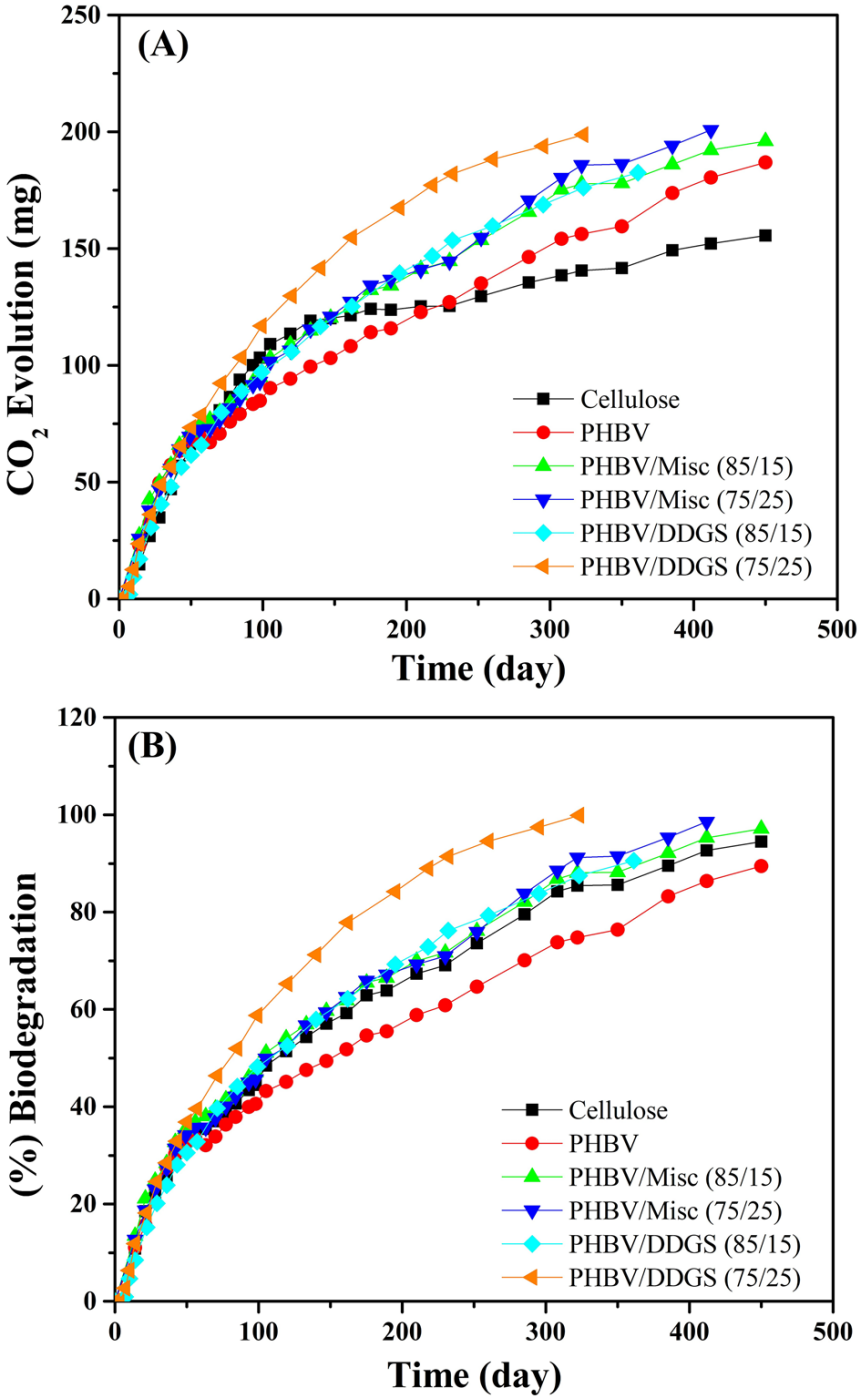
(**A**) CO_2_ evolution and (**B**) overall biodegradation for cellulose, PHBV, PHBV/Misc and PHBV/DDGS biocomposites.

Marine biodegradation of cellulose, PHBV/Misc and PHBV/DDGS biocomposites was completed and supports the hypothesis that inclusion of natural fibres and fillers from the biosphere into PHBV matrix will improve the biodegradation rate. Figure 4A,B indicate that PHBV and PHBV/Misc biocomposites have greater CO_2_ evolution compared to cellulose due to their 12% higher carbon content. Furthermore, the initial degradation rate of cellulose and PHBV/Misc biocomposites was metabolically similar due to the quantity of CO_2_ produced, which was attributed to the presence of cellulose and hemicellulose in Misc. The evolution of CO_2_ from PHBV has a consistent rate throughout biodegradation and was slower than for both composites and cellulose.

DDGS inclusion in PHBV is shown to improve the rate of biodegradation by a significant extent, clearly exceeding PHBV by day 50. Furthermore, after day 50, PHBV/DDGS (75/25) CO_2_ evolution exceeds cellulose. PHBV and PHBV/DDGS samples have approximately 20–25% higher theoretical maximum CO_2_ evolution relative to cellulose (see Supplementary Table S6), resulting in a greater quantity of CO_2_ being evolved. Elemental analysis results indicate a significantly higher nitrogen content in DDGS composites than expected, which will significantly promote microbial growth through protein synthesis. The significant presence of protein in PHBV/DDGS biocomposites implies that a nitrogen source will be readily available as either protein, thermally degraded protein or protein degradation products such as ammonia^21^. PHBV/Misc biocomposites hold no similar advantage for microbial population growth.

Cellulose biodegradation is shown to take a longer period of time in marine biodegradation when tested by ASTM D6691^40^. Different inoculums can provide different diversity of microorganisms with cellulolytic capabilities. Furthermore, soil/sand provides a greater microbiome that can accelerate biodegradation relative to marine water alone^13^. PHBV with 5–12% hydroxyvalerate marine biodegradation has been tested in accordance with ASTM D6691, but at a temperature of 30 °C^41^, such that the temperature enhanced the polymer enzymatic interactions and the overall biodegradation. PHBV biodegradation at 25 °C using this standard is the first of its kind and not comparable to any available literature results from other marine biodegradation standards. PHBV in this study biodegraded by 89.42% in 450 days.

Ramsay et al. described that 25% incorporation of starch in PHBV resulted in a 50% increase in the (%) biodegradation over a period of 32 days, however, this can be a characteristic of the high hydrophilicity and degradability of starch^19^. DDGS contains approximately 5% starch, the remaining constituents being mainly protein, cellulose, hemicellulose and lignin^33^ which do not have high hydrophilicity like starch. There are no studies of PHA biocomposites with only natural fillers/fibres in marine biodegradation at this time.

The extent of PHBV/Misc (85/15) and (75/25) biocomposites biodegradation is 15 and 25% higher compared to virgin PHBV in the same frame of time. This level of improvement is significantly lower compared to reported literature exposure in other forms of biodegradation medium, where a 100% improvement can be seen with 10–20% natural fibre loadings^15^. However, such studies were carried out with whole fibres, where the morphology of the fibre has significant importance, in addition to the different environment. 10% DDGS incorporation in PHA resulted in a 5–6 times improvement in initial mass loss and performed better than a 10% starch loading in soil^17^. PHA/DDGS (80/20) is also reported to compost 47–70% faster than PHA/Lignin, PHA/Jute, PHA/Lyocell and PHA/Hemp (80/20) in home composting conditions^16,42^. However, all studies on PHA/DDGS biodegradation follow no repeatable standards.

PHBV/DDGS (85/15) and (75/25) biocomposites biodegradation extent was approximately 15 and 40% higher than PHBV by day 295, and PHBV/DDGS (75/25) exceeds the biodegradation rate of cellulose after 150 days (see Supplementary Table S6). Furthermore, PHBV/DDGS (75/25) biocomposites exceed PHBV/Misc biocomposite biodegradation rate by day 40, and the biodegradation extent after 295 days is 18% higher. It takes 412 days for PHBV/Misc (75/25) biocomposites to biodegrade by ~ 97%, while PHBV/DDGS (75/25) biocomposites take 295 days. The final 2–3% carbon is expected to be used for cell growth and replication, resulting in less CO_2_ evolution. Composite biodegradation involves the concurrent degradation of filler and matrix, and DDGS composites PHBV fraction has a similar crystallinity to Misc composites, slightly above virgin PHBV. Therefore, it is hypothesized that there must be beneficial attributes of DDGS in biodegradation that exceed Misc.

Biodegradation is a complex process depending on the environment and the polymer or composite properties. The thermal properties of PHBV remained relatively unchanged, indicating the effects of the fibres on biodegradation are related to their composition and morphology, and that there is no expected improvement in the amorphous fraction of PHBV by DDGS or Misc to enhance biodegradation processes. Misc fibres do benefit more than DDGS from breaking of the biocomposite samples, as exfoliated Misc fibre is exposed in addition to channels from Misc fibre pull-out, increasing the exposed surface area, which may improve real world biodegradation processes. However, DDGS is physically a multi-layered grain-based material, easing fragmentation which may improve biodegradation rates in ways Misc fibre cannot.

Fundamentally, unlike Misc grass, DDGS has no lignin^33^, which is reported to be difficult to bioassimilate, unless in fermentative conditions. Furthermore, protein provides a suitable nitrogen source, which may already be partially degraded due to composite processing, and can be used for cell replication and growth in a local area that enhances DDGS biodegradation and assimilation^43^. The final aspect is the starch, which can be partially amorphous, and it is well documented that amorphous starch biodegrades faster than crystalline forms and by a wide variety of microorganisms^44^. Thus, PHBV biocomposites with high DDGS loadings have faster biodegradation rates than PHBV/Misc biocomposites in a similar time frame, provided there is enough DDGS incorporated. In conclusion, proteinaceous DDGS enhances biodegradation better relative to Misc.

Therefore, to implement sustainable, low-cost and lightweight plastic technologies in industry, enhancing PHBV with Misc and DDGS can improve the mechanical and biodegradable properties, respectively, which can increase the utility of biodegradable plastics and reduce the future build-up of plastics in oceans. Protein based sustainable fillers, such as DDGS, are the better option to improve biodegradation rates of plastics compared to other non-proteinaceous natural fillers and fibres. Furthermore, their use increases the value chain of the agri-food industry and the green character and sustainability of biocomposites.

## Materials and methods

### Materials

Poly(3-hydroxybutyrate-co-3-hydroxyvalerate) (PHBV) pellets (ENMAT Y1000P), with 1–5% 3-hydroxyvalerate, were purchased from Tianan Biological Materials Co. Ltd., Ningbo City, Zhejiang Province, PR China. *Miscanthus* (Misc) grass fibres were donated by the New Energy Farms, Ontario, Canada, and had an average length and diameter of 4.65 ± 2.5 and 0.74 ± 0.02 mm, respectively. Distillers’ dried grains with solubles (DDGS) was donated from Integrated Grain Processors Co-operative Inc. (IGPC), Aylmer, Ontario, Canada and had an average size of 0.36 ± 0.18 mm. It was washed with tap water for 15 min at room temperature following the methodology outlined by Zarrinbakhsh et al.^20^. Marine sediment and marine water were kindly provided from the rainbow reef exhibit by Ripley’s Aquarium in Toronto, Canada. Barium hydroxide (BaOH_2_) and hydrochloric acid (HCl) were purchased from Fisher Scientific and LabChem, respectively. 25-(Bis(5-tert-butyl-2-benzo-oxazol-2-yl) thiophene (BBOT), cellulose filter paper and diiodomethane used for characterization were purchased from Thermo Fisher Scientific.

### Composite processing

Prior to processing Misc fibre, DDGS and PHBV pellets were kept overnight at 80 °C. Composite processing was completed in a DSM Explore (DSM research, Netherlands), co-rotating twin screw with a processing temperature of 180 °C, a fill pack and hold of 10 MPa, and a 2-min retention time at 100 rpm. PHBV/DDGS and PHBV/Misc biocomposites were produced in ratios of (85/15 wt%) and (75/25 wt%).

### Elemental analysis (CHNS)

Elemental analysis (C, H, N and S) of cellulose, PHBV, PHBV/Misc, PHBV/DDGS and marine sediment samples was completed using a Perkin-Elmer elemental analyser Flash 2000 (Thermo Scientific, USA). The column was heated to 950 °C with carrier gases of helium and oxygen at flow rates of 140 and 250 mL/min, respectively. Samples between 2 and 3 mg were taken and 4 replicates were completed for each material, while 6 BBOT samples were measured to ascertain a calibration curve.

### Thermal properties

#### Differential scanning calorimetry (DSC)

PHBV and its composites were analysed by DSC (Q200, TA instruments, Delaware, USA). Samples weighing 5–10 mg were heated at 10 °C/min from − 40 to 200 °C. The cooling cycle was at 10 °C/min to − 40 °C, with isothermal conditions lasting 3 min, followed by reheating. The melting temperature (T_m_) and enthalpy of melting (ΔH_m_) were observed from the first and second heating cycles, respectively.

The % crystallinity (${X}_{C}$) of PHBV in the polymer composites was calculated from first and second heating cycles using Eq. (1).

1 $${X}_{C}=\left(\frac{{\Delta H}_{m}}{{\Delta H}_{m}^{0}\times {w}_{f}}\right)\times 100 \%$$

where ${\Delta H}_{m}^{0}$ is the enthalpy of 100% crystalline PHBV (109 J/g)^45^ and ${w}_{f}$ belongs to the weight fraction of PHBV.

#### Thermogravimetric analysis (TGA)

TGA analysis (Q500, TA instruments, Delaware, USA), was completed on 15–20 mg samples of virgin PHBV, *Miscanthus*, DDGS and PHBV biocomposites. A heating rate of 10 °C/min to 700 °C was applied under a N_2_ atmosphere. Purge and balance flow rates of 40 and 60 ml/min were used. The ash content of the marine sediment, cellulose, PHBV and PHBV/Misc and PHBV/DDGS composites was analysed in air at 900 °C, in accordance with ASTM D2584.

### Mechanical properties

#### Tensile and flexural properties

The samples were conditioned in accordance with ASTM D618 for 48 h. Characterization of mechanical properties was completed with an Instron 3382 (Massachusetts, USA). Type IV tensile samples were analysed at 5 mm/min according to ASTM D638. Flexural samples were tested following ASTM D790 with a span of 52 mm. 5 replicates of each sample were tested.

#### Notched IZOD impact properties

Notched IZOD impact samples were tested using a Zwick Roell HIT25P (Ulm, Germany) after conditioning of samples according to ASTM D618. The sample was tested following ASTM D256 and an average value of six replicates was considered as the impact strength.

### Scanning electron microscopy (SEM)

Impact fractured samples were analysed by SEM using a Phenom ProX desktop (Eindhoven, Netherlands). All samples were gold sputter coated for 10 s by a Cressington Sputter Coater (Watford, England), and analysed using 10 kV accelerating voltage. Samples were analysed under 1000 × magnification.

### Contact angle

The contact angle of PHBV and composites were determined using a Ramé-Hart goniometer 260-U1. Utilizing the sessile drop technique, deionized water or diiodomethane was dropped onto the surface of the injection moulded samples, functioning as polar and non-polar testing liquids. DROPimage software was utilized to anlyse the relative surface contact angles of three replicates for each sample to determine the hydrophilicity of the neat polymer and composites.

### Marine biodegradation

Marine biodegradation was completed in accordance with ASTM D7991-15. A ratio of 150 g of marine water to 250 g of sediment was used, and 100 mg of cryoground cellulose (positive blank) and the test samples (PHBV and PHBV-based composites) were submersed in the sediment. All tested samples were in powder form. The cellulose was used as standard material to observe the validation criteria of marine biodegradation experiment. The marine water and sediment were used within 3 days of acquisition. The environment chamber was maintained at 25 ± 2 °C. To ensure an airtight seal on the desiccators, petroleum jelly and parafilm were used at the periphery of the lid after each titration. Desiccators were gently shaken every day to break the CaCO_3_ layer that developed from the reaction of BaOH_2_ and CO_2_ in the beaker. The initial conditions of the marine water over an entire month before acquisition are outlined in Table 1. The sediment total organic carbon (TOC) is 0.09%.

**Table 1:** Marine water initial parameters.

Temp (°C)	pH	Salinity (µg/L)	NH_3_-N (mg/L)	NO_2_-N (mg/L)	Dissolved Oxygen (mg/L)	Alkalinity (mg/L CaCO_3_)	Nitrate (mg/L)	TOC (mg/L)
23.55	8.06	31.9	0.01	0.004	7.5	164	35	1.8

## Supplementary information

Supplementary Information.

## Abbreviations

DDGS: Distillers’ dried grains with solubles
Misc: *Miscanthus*
PHA: Poly(hydroxyalkanoate)
PHBV: Poly(3-hydroxybutyrate-co-3-hydrovalerate)
PHB: Poly(3-hydroxybutyrate)
PH4B: Poly(3-hydrobutyrate-co-4-hydroxybutyrate)
PLA: Poly(lactic acid)
