# Supplementary material for: The effect of natural fillers on the marine biodegradation behaviour of poly(3-hydroxybutyrate-co-3-hydroxyvalerate) (PHBV)
Source: Sci Rep. 2021 Jan 13;11:911. doi: 10.1038/s41598-020-78122-7 (PMC7806601; doi:10.1038/s41598-020-78122-7)
Supplement: Supplementary file 1 — Supplementary Information. [file 41598_2020_78122_MOESM1_ESM.docx]

**Supplementary Materials**

**The Effect of Natural Fillers on the Marine Biodegradation Behaviour of Poly(3-hydroxybutyrate-co-3-hydroxyvalerate) (PHBV)**

*Kjeld W. Meereboer^1,2^, Akhilesh K. Pal^2^, Erick O. Cisneros-López^1^_,_ Manjusri Misra^1,2*^ and Amar K. Mohanty^1,2*^*

*^1^School of Engineering, Thornbrough Building, University of Guelph, 50 Stone Road East, Guelph, Ontario*

*^2^Bioproducts Discovery and Development Centre, Department of Plant Agriculture, Crop Science Building, University of Guelph, 50 Stone Road East, Guelph, Ontario*

***Corresponding Authors:** Amar K. Mohanty; Email: [mohanty@uoguelph.ca](mailto:mohanty@uoguelph.ca)

Manjusri Misra; Email: [mmisra@uoguelph.ca](mailto:mmisra@uoguelph.ca)

**Table S1:** PHBV and PHBV biocomposite mechanical properties.

| **Sample** | **Tensile Modulus** | **Tensile Strength** | **Flexural Modulus** | **Flexural Strength** |
| --- | --- | --- | --- | --- |
| **PHBV** | 3.78 ± 0.15 | 42.6 ± 1.2 | 3.74 ± 0.25 | 70.5 ± 2.5 |
| **PHBV/Misc 85/15** | 5.89 ± 0.33 | 43.1 ± 0.7 | 5.61 ± 0.10 | 76.4 ± 1.1 |
| **PHBV/Misc 75/25** | 7.66 ± 0.94 | 43.9 ± 0.9 | 6.50 ± 0.44 | 74.1 ± 3.5 |
| **PHBV/DDGS 85/15** | 3.68 ± 0.27 | 28.9 ± 0.5 | 3.53 ± 0.10 | 56.6 ± 1.1 |
| **PHBV/DDGS 75/25** | 3.50 ± 0.10 | 23.3 ± 0.3 | 3.32 ± 0.03 | 45.7 ± 0.8 |

**Table S2:** DSC of PHBV and its biocomposites.

|  | **First Heating Cycle** | | | **Second Heating Cycle** | | |
| --- | --- | --- | --- | --- | --- | --- |
|  | **T_m1_ (°C)** | **∆H_m1_ (J/g)** | **X_c1_ (%)** | **T_m2_ (°C)** | **∆H_m2_ (J/g)** | **X_c2_ (%)** |
| **PHBV** | 176.19 ± 3.99 | 75.06 ± 1.69 | 68.86 | 173.84 ± 0.83 | 78.94 ± 0.66 | 72.42 |
| **PHBV/Misc 85/15** | 174.66 ± 1.51 | 63.61 ± 2.85 | 68.65 | 168.76 ± 0.10 | 68.81 ± 0.30 | 74.27 |
| **PHBV/Misc 75/25** | 171.13 ± 0.38 | 57.74 ± 5.32 | 70.62 | 168.26 ± 0.91 | 62.55 ± 4.10 | 76.51 |
| **PHBV/DDGS 85/15** | 174.07 ± 2.67 | 66.24 ± 0.93 | 71.49 | 169.79 ± 0.19 | 73.70 ± 1.97 | 79.54 |
| **PHBV/DDGS 75/25** | 172.23 ± 2.52 | 53.63 ± 1.05 | 71.72 | 168.98 ± 0.15 | 66.78 ± 2.21 | 81.68 |

**Table S3:** TGA of marine biodegradation samples, miscanthus, DDGS and cellulose.

| **Sample** | **Temperature at 10% Mass Loss (°C)** | **Peak Degradation Temp. (°C)** | | **Ash Content (%)** |
| --- | --- | --- | --- | --- |
| **Cellulose** | - | - | | 0.00 |
| **PHBV** | 286.86 | 302.99 | | 1.59 ± 0.08 |
| **PHBV/Misc 85/15** | 273.67 | 292.85 | 354.42 | 1.43 ± 0.13 |
| **PHBV/Misc 75/25** | 273.73 | 284.28 | 355.95 | 1.81 ± 0.29 |
| ***Miscanthus* Fibre** | 284.74 | 358.16 | | 1.49 ± 1.03 |
| **PHBV/DDGS 85/15** | 282.44 | 297.47 | | 1.70 ± 0.25 |
| **PHBV/DDGS 75/25** | 267.95 | 287.08 | 350.97 | 1.94 ± 0.10 |
| **DDGS** | 267.52 | 288.73 | 348.94 | 3.25 ± 1.58 |
|  |  |  | |  |
| **Sediment** | N/A | N/A | | 59.15 ± 0.30 |

**Table S4:** Elemental analysis of marine biodegradation samples and sediment.

| **Sample** | **Carbon (%)** | **Hydrogen (%)** | **Nitrogen (%)** | **Sulphur (%)** | **H/C^a^** |
| --- | --- | --- | --- | --- | --- |
| **Cellulose** | 43.94 ± 1.17 | 6.40 ± 0.16 | 0.05 ± 0.00 | 0.00 ± 0.00 | 1.73 |
| **PHBV** | 56.93 ± 1.77 | 7.05 ± 0.10 | 0.46 ± 0.06 | 0.00 ± 0.00 | 1.48 |
| **PHBV/Misc 85/15** | 55.04 ± 0.28 | 6.94 ± 0.04 | 0.55 ± 0.01 | 0.00 ± 0.00 | 1.50 |
| **PHBV/Misc 75/25** | 55.55 ±0.64 | 6.79 ± 0.08 | 0.54 ± 0.04 | 0.00 ± 0.00 | 1.46 |
| **PHBV/DDGS 85/15** | 54.83 ± 0.13 | 5.97 ± 0.71 | 6.66 ± 0.03 | 0.00 ± 0.00 | 1.30 |
| **PHBV/DDGS 75/25** | 54.16 ±0.09 | 5.25 ± 0.41 | 7.64 ± 0.76 | 0.00 ± 0.00 | 1.16 |
|  |  |  |  |  |  |
| **Sediment** | 11.20 ± 0.13 | 0.10 ± 0.00 | 0.00 ± 0.00 | 0.00 ± 0.00 | 0.11 |

^a^ Molar Ratio of hydrogen to carbon.

**Table S5:** Contact angles of polymer composites and cellulose filter paper.

| **Contact Angle (deg)** | | |
| --- | --- | --- |
| **Polymer/Composite** | **Deionized Water** | **Diiodomethane** |
| **Cellulose** | 00.0 ± 0.0 | 00.0 ± 0.0 |
| **PHBV** | 66.7 ± 1.8 | 39.1 ± 2.6 |
| **PHBV/Misc 85/15** | 73.1 ± 0.4 | 42.8 ± 1.6 |
| **PHBV/Misc 75/25** | 73.0 ± 1.8 | 44.4 ± 0.6 |
| **PHBV/DDGS 85/15** | 66.6 ± 1.2 | 39.7 ± 1.6 |
| **PHBV/DDGS 75/25** | 70.1 ± 0.9 | 37.3 ± 1.5 |

**Table S6:** Marine biodegradation results.

| **Sample** | **CO_2_ Evolved (mg)** | **Theoretical CO_2_ (mg)** | **Biodegradation (%)** | **Time (days)** |
| --- | --- | --- | --- | --- |
| **Cellulose** | 152.15 | 155.60 | 96.38 | 450 |
| **PHBV** | 180.44 | 186.85 | 89.42 | 450 |
| **PHBV/Misc 85/15** | 192.27 | 190.70 | 94.48 | 450 |
| **PHBV/Misc 75/25** | 204.73 | 200.78 | 98.58 | 412 |
| **PHBV/DDGS 85/15** | 175.76 | 182.448 | 90.57 | 361 |
| **PHBV/DDGS 75/25** | 206.72 | 193.87 | 97.42 | 295 |
